# Biosimilars in rheumatology: recommendations for regulation and use in Middle Eastern countries

**DOI:** 10.1007/s10067-018-3982-9

**Published:** 2018-02-06

**Authors:** Bassel El Zorkany, Nizar Al Ani, Samar Al Emadi, Jamal Al Saleh, Imad Uthman, Yasser El Dershaby, Mohamed Mounir, Hani Al Moallim

**Affiliations:** 1 Rheumatology Department, Cairo University, Cairo, Egypt; 2 Rheumatology Unit, Baghdad College of Medicine, Medical City, Baghdad, Iraq; 3 Hamad Medical Corporation, Doha, Qatar; 4 Rheumatology Unit, Dubai Hospital, Dubai Health Authority, Dubai, United Arab Emirates; 5 Division of Rheumatology, American University of Beirut Medical Center, Riad El-Solh, Beirut, Lebanon; 6 Pfizer Africa and Middle East, Dubai, United Arab Emirates; 7 Medical College, Umm Alqura University (UQU), Mecca, Saudi Arabia

**Keywords:** Biosimilar, Intended copies, Middle East, Recommendations (TBC), Rheumatology, Switching

## Abstract

The increasing availability of biosimilar medicines in Middle Eastern regions may provide an opportunity to increase the number of rheumatology patients who have access to traditionally more expensive biologic medicines. However, as well as a lack of real-world data on the use of biosimilar medicines in practice, the availability of intended copies in the region may undermine physician confidence in prescribing legitimate biosimilar medicines. There is a need for regional recommendations for healthcare professionals to ensure that biosimilar drugs can be used safely. Therefore, a literature search was performed with the aim of providing important recommendations for the regulation and use of biosimilar medicines in the Middle East from key opinion leaders in rheumatology from the region. These recommendations focus on improving the availability of relevant real-world data, ensuring that physicians are aware of the difference between intended copies and true biosimilars and ensuring that physicians are responsible for making any prescribing and switching decisions.

**Electronic supplementary material:**

The online version of this article (10.1007/s10067-018-3982-9) contains supplementary material, which is available to authorized users.

## Introduction

Middle Eastern countries have recently seen an increase in demand for healthcare services, in part due to increased income in the region and better access to modern amenities leading to a surge in lifestyle-related illnesses [1]. Several member countries of the Gulf Cooperation Council (GCC) have therefore been making significant investments in healthcare: for example, in Saudi Arabia in 2014, $28.8 billion was allocated to health and social welfare, including 11 new hospitals, 11 medical centers, and two medical complexes in addition to 132 hospitals and centers under construction [2].

On the other hand, the quality of healthcare provision, particularly in the field of rheumatology, varies across the region due to differences in allocated government resources, patient demographics, the number of qualified rheumatologists, political stability, and population movements [3]. In some regions of the Middle East, patients cannot access the care they require [1].

In recent years, biologics have significantly altered the management and outcomes of rheumatoid arthritis (RA) [4].

Biologics are complex and variable molecules produced in living cells using recombinant DNA technology. Their manufacture is a challenging process and can have profound clinical implications—small changes in the manufacturing process can have a large impact on the clinical profile of the final product [5, 6]. Therefore, any changes to the process are evaluated by regulators to ensure they do not affect the safety and efficacy of the products [7]. A comparability assessment provides evidence that the efficacy and safety of the product after a manufacturing change are within the parameters of the product prechange [8].

The efficacy and safety profiles of many biologics have been extensively studied and are well established in patients with RA [9]. However, due to the high costs associated with these therapies, they can become a significant financial burden in many world markets, including some countries of the Middle East [6].

On the other hand, early access to biologics in Saudi Arabia and other GCC countries is more prevalent than other parts of the world due to strong financial support from governments [10]. There is significant delay in initiating disease-modifying anti-rheumatic drugs (DMARDs) and biologic therapy worldwide [11]. The introduction of biosimilars may help reduce the costs of biologic therapies, therefore potentially enabling a reduction in the delay to biologic treatment [12].

For many biologics, the worldwide patent expiry date has already passed or will pass in the near future. This, coupled with the high costs of biologics, provides an opportunity for biosimilar medicines to enter many markets, especially where access to biologics is particularly difficult due to costs [6, 13]. However, the barrier to uptake might be that rheumatologists need more clinical data, particularly in the real-world setting, to convince them that biosimilars are equally as safe and effective as originator drugs [6].

The present study was performed to review the current medical literature for available evidence on the roles of biosimilars in patients with RA, with the aim of informing a comprehensive set of consensus recommendations for biosimilars use in the Middle East. Information pertinent to the use of biosimilars in clinical practice will be discussed briefly, namely clinical trial results, regulatory approvals, and product labels with a focus on Middle Eastern countries.

Current documents with recommendations for countries of the Middle East region, where they exist, are listed in Online resources.

## Methods

### Literature search

A literature search for relevant papers on biosimilars in rheumatology was performed. A PubMed search from January 2010 to June 2017 was conducted using relevant search terms (available in Online resource 3). This was followed by a search of American College of Rheumatology (ACR) meeting abstracts (2014–2016) and accepted European League Against Rheumatism (EULAR) meeting abstracts (2015–2017) using the same search terms. Articles were restricted to English language publications and studies conducted in human subjects. The search was supplemented with a hand search through the websites of several Middle East societies for any pertinent information on biologics or biosimilars.

The findings of the literature search were then analyzed by a panel of key opinion leaders from the Middle East region as part of an international online webcast discussion.

## Results

### Literature search

**Fig. 1:**
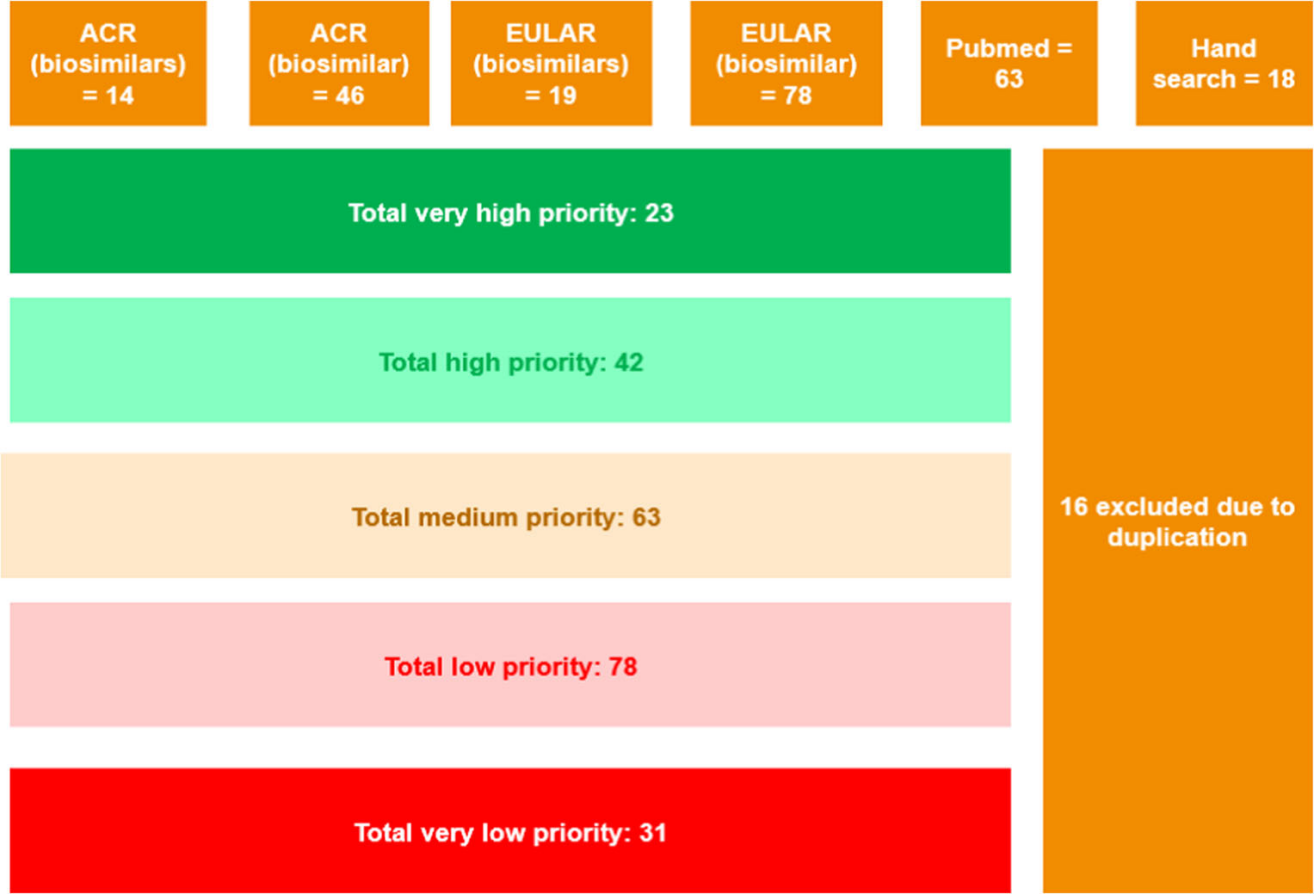
An analysis of the results of the literature search

In all, a total of 222 articles were identified. Of these, 81 publications and a further 157 abstracts from EULAR and ACR annual meetings were selected for detailed review (Fig. 1). Priority was determined based on relevance to clinical practice and switching.

### Key themes

Based on the results of the literature search, a number of key themes relating to the regulation and use of biosimilars were identified: approval and regulation, pharmacovigilance, use in clinical practice, and switching. These themes are presented in detail below.

### Biosimilar medicines

A biosimilar medicine is a copy of a biologic medicine, which is described as a “reference biologic” or “reference product.” Because biosimilar manufacturers do not have access to the details of the manufacturing processes of reference biologics, and therefore, manufacturing is based on a process of reverse engineering, minor differences may exist between the final products [6, 8, 13]. Indeed, it is unfeasible to create an exactly identical copy using a reference biologic, because of the complexity of the manufacturing process, the heterogeneity of the final product (antibody-based therapies in the case of RA), in-process controls and product controls, impurities, aggregates, fragments, and other factors. This means that a biosimilar cannot be described as a generic medicine. Hence, the biosimilar manufacturer must provide data demonstrating that the minor differences between the biosimilar and its reference product do not affect its safety or efficacy [6, 14].

Because of the above considerations, granting marketing authorization in many countries is subject to stringent regulations and quality control analyses set by international guidelines from WHO, the European Medicines Agency (EMA), or US Food and Drug Administration (FDA) [6]. Physicochemical and functional analyses, together with animal or early phase clinical studies, are used in a stepwise manner to confirm that the biosimilar product demonstrates the same safety and efficacy data as its reference product [6]. Thereafter, an appropriate head-to-head clinical trial should be conducted to compare the biosimilar to its reference product [15]. In 2013, an infliximab biosimilar, CT-P13, was the first monoclonal antibody biosimilar approved in the EU based on an extensive nonclinical and clinical comparative data package [16]. Regulatory pathways in some Middle Eastern countries are not yet established.

In recent years, over 20 biosimilar products have come to market globally including ABP-501 (adalimumab), CT-P13 (infliximab), and SB4 (etanercept) [17–19]. A list of biosimilars of biologics currently available for the treatment of inflammatory diseases for which data have been published in peer-reviewed journals or presented at international scientific meetings is available in Online resources (Table 2). Currently, more than 700 biosimilar products have been reported to be in preclinical and clinical trials for various conditions [17].

However, not all versions of biologics have evidence to demonstrate biosimilarity [20]. These medicines are referred to as “intended copies” [20]. Intended copies of biologics can be defined as copies of already licensed biologic products that have not met requirements to establish biosimilarity as set out by the WHO, EMA, or FDA [14]. Biosimilars are required to undergo a stepwise regulatory process to ensure similarity and clinical equivalence to the reference product, whereas intended copies are marketed products that have escaped this process [21]. Therefore, intended copies may pose significant safety risks for patients. In the Middle East region in particular, intended copies are of important concern because the pharmaceutical market is open, which means it is difficult to track the number of drugs available, including those which are unapproved or have been brought into the region illegally. There are currently two known intended copies available in Iraq (see Online resource 1).

An observational study performed in Mexico and Colombia found that a significant percentage (14.3%) of patients receiving Infinitam/Etanar or Kikuzubam (intended copies of etanercept and rituximab, respectively) experienced grade 3/4 adverse events within a very short time of starting treatment, including 36.2% of affected patients who experienced adverse events on the same day as treatment [22]. Kikuzumab was later withdrawn from the market but Infinitam is still available [17].

### Biologics in the Middle East

For RA specifically, there is suboptimal use of biologic therapies across the Middle East and North Africa region, with fewer than 2% of RA patients receiving treatment with antitumor necrosis factor drugs in some countries, compared with 40% of patients in the USA. This is mainly due to high associated costs and the fact that this is considered a low priority condition in the region despite its widespread prevalence [23, 24]. A panel of local rheumatologists convened in 2013 to discuss implementation of EULAR recommendations in the Middle East. The low use of biologics in the region may be due to drug access, monitoring issues, and development of tuberculosis in endemic populations [23]. In some countries, including Lebanon, regulatory pathways for approval exist only in draft form [25].

However, one single-center registry of RA in Jeddah, Saudi Arabia, has around 520 patients, approximately 20% of whom are treated with biologics (unpublished experience), so the proportion of patients treated with these medicines may vary considerably across the region [26].

### Approval and regulation in Europe and USA

Legislation and guidelines from regulatory agencies, such as the FDA and EMA, and WHO have been developed because the approval process of biosimilars cannot follow the same guidance as generics due to their complexity [21, 27]. Although most countries have adopted these guidelines for local approval of biosimilars, others are not as stringent with their regulations [21].

Developing a biosimilar comes at a similar cost to developing the bio-originator. Therefore, the primary driver of cost savings may arise from a reduced requirement for studies to prove efficacy and safety directly [28]. It is notable that the entire clinical program of the reference product does not need to be replicated for approval of the biosimilar for the same indications as the reference product because the guidelines imply that efficacy and safety data can be extrapolated from the reference product’s clinical studies for the other indications [20], if the mechanism of action is not fully understood or may differ between indications. This eliminates the need for multiple trials of the biosimilar in various indications, which can decrease costs and help expedite access to the medicine. According to the guidelines, the decision to grant approval in multiple indications should be evaluated on a case-by-case basis and depends on the scientific evidence presented [14, 29, 30].

However, given the complex nature of biologic drugs, several medical societies have expressed concerns regarding extrapolation [6, 31–35]. Where data have been extrapolated for additional indications at the approval phase, real-world monitoring and postapproval measures are particularly important. Physicians may be cautious about prescribing for indications that have not specifically been investigated during the approvals phase.

#### Approval pathways for biosimilars in other regions

Most other countries outside the USA and Europe have adopted the guidelines set up by the FDA, EMA, or WHO [27]. In Mexico, for example, access to biologics is a complex process, which assumes that safety and efficacy data relating to a biosimilar cannot be extrapolated or considered interchangeable to the reference product [36].

In Latin America, most countries have adopted the WHO recommendations, although some countries have established their own guidance for biosimilars [37]. Products classified as intended copies exist in Latin America, many of which were approved without adequate evaluation or quality clinical trials before appropriate regulations for biosimilars were introduced [15]. Recent legislation has stipulated that these intended copies should be subject to the same regulations as biosimilars and a complete assessment of these products is expected to take 2 years from the effective date of the new regulations [20]. However, re-evaluating intended copies that were previously approved and now do not fit within each country’s regulatory criteria for biosimilars remains a challenging issue in Latin America and many developing countries [20].

In Asia, many countries have already established or are establishing regulatory guidance for the use of biosimilars [20]. Furthermore, as per Latin America, many “similar biologics” were already approved in India before the introduction of regulatory guidelines [20]. Registration and traceability of intended copies in India and China as well as other Asian countries remains a challenge.

In the Middle East region, each country has a different health system with its own laws concerning registration of new drugs [25], and recently, some countries have established their own regulatory frameworks for biosimilar approval (see Online resource Table 1) either by adopting the EMA guidance or developing their own framework [24, 38]. For instance, the guidance issued by the Saudi Food and Drug Authority is adapted from the EMA guidance [39]. The guidance states that an extensive comparability exercise is required to demonstrate that a biosimilar has a highly similar quality profile when compared to the chosen or selected reference medicinal product [38]. On the other hand, the Iranian National Regulatory Authority (NRA) based its guidance on the WHO guidelines for biosimilars, although there are clear differences between these two documents and the Iranian guidance is not as stringent as that from WHO [36].

The Lebanese Ministry of Public Health (MOPH) has developed guidance to regulate these drugs [40]. The guidance is largely based on EMA, WHO, and the French National Agency for Medicines and Health Products safety guidelines [25]. The current laws and guidance in Lebanon, including the “General Regulatory and Manufacturing Rules” document from the Ministry of Public Health, only endorse the use of biosimilars that have been approved by the FDA or EMA and do not violate pharmaceutical patent laws [41].

Despite the variance in guidance and approval pathways for biosimilars in the Middle East region, an important consideration is the existence and availability of intended copies in the region. Domestic intended copies referred to as “copy biologics” or “biogenerics” are available in some countries, for example Iran. These medicines were licensed in Middle East countries before the introduction of formal regulatory guidance (equivalent to international standards) [24]. The presence of these intended copies in the region along with pharmacovigilance practices that are very much less stringent than those in Europe and the USA poses a significant challenge to optimal use of biologics or biosimilars in clinical practice. Limited real-world data on intended copies are available [42].

### Pharmacovigilance

Clinical studies of biosimilars conducted in relatively small numbers of patients over short time periods are not powered to give a full picture of the long-term safety profile of these products when used in the real world. Therefore, regulatory submission must include a robust pharmacovigilance program to record rare adverse events with the biosimilar, especially related to immunogenicity [43]. Immunogenicity, the ability of a biologic to elicit an immune response, is a significant concern with these medicines, and predictors of aggregation underlying immunogenicity are incompletely understood [28, 44].

The EMA, FDA, and WHO guidelines stipulate provision of a plan for pharmacovigilance by the manufacturer as well as a risk management plan similar to that for the reference biologic when seeking biosimilar marketing approval [14, 29, 30].

Effective pharmacovigilance is only possible if biologics or biosimilar medicines are traceable so that specific adverse events may be assigned to a particular product or batch. The EMA, WHO, and FDA recommend that all national regulatory agencies develop a framework for effective supervision of pharmacovigilance and traceability of biosimilars [14, 29, 30]. To achieve this, systems to identify the product by brand name and batch number are required [20, 45]. Therefore, appropriate naming of biosimilars is essential [14, 31, 45].

The WHO states that any reports of adverse reactions of any biologic product should include the international common denominator (ICD), an identifier assigned to all biologics, the product/brand name, manufacturer name, lot number, and country of origin [30]. The EMA guidance states that a patient’s medical file, managed by the physician, should contain information on any substitution of a biologic with a biosimilar and include the brand name [29, 31]. However, naming of biosimilars is yet to be standardized internationally [43].

A recent position paper on biosimilars in Latin America called for an improvement in pharmacovigilance and an improved process to trace adverse events related to biosimilars, and identified a need for better systems to capture and analyze safety data [37].

In 2015, the Egypt Ministry of Health initiated a large pharmacovigilance department, which can request that pharmaceutical companies provide further data in particular areas, including conducting new studies in local conditions. This may impact regulatory circumstances for the approval and monitoring of biosimilars [46].

### Lack of physician confidence in biosimilars

There seems to be a perception among patients and physicians that biosimilars are less safe or effective than their reference products [47]. Because of the availability of intended copies in the Middle East, there is a tendency for physicians to be wary of legitimate biosimilar medicines by association. Indeed, many physicians in the Middle East region are reluctant to prescribe biosimilars despite their competitive pricing probably because of a lack of confidence in the efficacy or safety profiles of these medicines [25, 41]. In a survey of 117 physicians from Lebanon, Egypt, Syria, Algeria, Iraq, Sudan, Jordan, Iran, Belgium, and Italy, only 41% of responders stated that they prescribe biosimilars and, in most cases, only drugs that have been approved by the FDA and EMA [25].

In Iraq, intended copy medicines are usually only used if there is no alternative available. The use of intended copies in rheumatology is still relatively new in comparison to other specialties, with the only experiences from Iraqi patients who reported receiving these treatments from other countries, such as India.

The publication of national rheumatology guidelines on prescribing biosimilars in the UK, Germany, Belgium, and other Western countries may have helped physicians gain confidence in prescribing “true biosimilars” [17, 47].

### Switching

Switching from one biologic agent to another can be performed for medical or nonmedical reasons [48]. The decision about whether to switch patients between a reference product and its biosimilar for nonmedical reasons remains a challenge for many clinicians worldwide. Guidelines specifically related to switching are limited [17].

Switching or even starting a biologic is certainly a challenge in the Middle East region. A survey of Arab physicians found that a lack of clear guidelines on interchangeability and substitutability with reference products may cause them to be more cautious in prescribing biosimilars until they are more confident regarding their efficacy and quality [25].

The Rheumatology Department at Baghdad Teaching Hospital has proposed a mechanism for treating rheumatoid arthritis with the Iranian intended copy (Zytux). This system prescribes the drug only for rituximab-naive patients with active disease or switching to Zytux with a minimum of 6 months washout period from the last rituximab dose, provided patients will not be switched again to originator rituximab.

The most important concerns about switching relate to loss of efficacy or emergence of a hypersensitivity reaction after switching [49]. Therefore, for clinicians to switch patients from a reference product to its biosimilar, factors such as the availability of clinical trial evidence and similarity with the reference drug as well as real-life evidence may be of key importance [50]. Indeed, decisions regarding switching should be based on evidence-based clinical data, where it exists, as well as clinician expertise. Randomized controlled trials, meta-analyses, and cohort observational studies provide especially useful data [51].

Switching decisions should be made by the treating physician with patients’ informed understanding and consent. The decision should be individualized for each patient based on risk factors, preferences, and individual patient characteristics, which should be taken into account by consulting with and educating the patient regarding a switch. Any therapeutic or switch decisions from biologic drug to a biosimilar, therefore, should be carried out on an individual basis and based on best available scientific evidence and evidence-based practice [31, 46].

#### Clinical studies of switching

There are many clinical studies demonstrating similar efficacy and safety of biosimilars compared with their reference products in both biologic-naive and switch patients with RA (see Online resource Table 3). Long-term safety and efficacy of SB4, a biosimilar of etanercept, were reported at the ACR 2016 annual meeting [49]. In this study, 245 patients with moderate to severe RA were randomized to either SB4 or reference etanercept, plus methotrexate (for both groups). The 52-week randomized double-blind period was followed by a 48-week open-label extension period and patients were followed up to week 100. The trial demonstrated that long-term efficacy was comparable between patients who continued on SB4 or switched to SB4 from etanercept during the extension period [52]. However, immunogenicity was assessed before and after the first dose but not after the transition, and so remains unknown. The effect of the switch on drug pharmacokinetics was also not assessed [53].

In a trial of SB5, an adalimumab biosimilar, patients with RA were randomly assigned to receive reference adalimumab or SB5 for 24 weeks, then those in the adalimumab arm were randomly assigned to continue reference adalimumab or transition to SB5 in an extension phase up to week 52. Comparable efficacy was seen across treatment groups at 52 weeks [54].

The above studies included only a single transition to the biosimilar agent from the reference product. However, it has been hypothesized that to establish comparable safety and efficacy of alternating between biologics and biosimilars definitively, at least two switches are required [55]. Therefore, some caution should be applied when interpreting the findings of studies that included only a single switch.

The infliximab biosimilar CT-P13 has been extensively studied using biosimilarity analyses of quality, safety, and efficacy to the reference drug in patients with ankylosing spondylitis (AS) (the PLANETAS study) and in patients with RA (the PLANETRA study) [16, 50, 56–59]. Both these studies demonstrated equivalent efficacy of CT-P13 to reference infliximab, with a comparable pharmacokinetic profile and immunogenicity as well as comparable safety profiles for up to 2 years after starting treatment with CT-P13 [16, 56–59].

To investigate equivalence between infliximab and CT-P13 further, the Norwegian government funded the NOR-SWITCH study. NOR-SWITCH was a 12-month, randomized, double-blind clinical trial to investigate maintenance of efficacy and monitor adverse events following a transition from reference infliximab to CT-P13 versus remaining on reference product. The study included 482 patients with inflammatory diseases, including 77 with RA, who had been on reference infliximab for at least 6 months [17, 60]. Disease worsening was chosen as a primary endpoint because the study was conducted in patients stable on reference infliximab and the endpoint had to be applicable across indications. Results across diagnoses were therefore heterogeneous, although definitions of disease worsening in each indication were based on established measures of disease activity. The NOR-SWITCH study demonstrated that switching from infliximab to CT-P13 was not inferior to continued treatment with reference infliximab, although the study was not powered to detect differences in individual indications [60].

In general, clinical studies of biosimilars have demonstrated adverse event profiles that are comparable to their reference products (see Online resource Table 3).

#### Real-world data for switching

Several studies have analyzed real-world outcomes of transitioning from reference compounds to biosimilars [17].

Real-world data for CT-P13 have also shown similar efficacy and safety as the clinical analyses [52, 61]. A single-center, observational study of 34 RA patients switched from originator infliximab to CT-P13 and followed for a mean 15.8 months showed comparable safety and efficacy before and after the switch. However, immunogenicity before and after the switch was not recorded and remains unknown [61]. Some real-world data indicate that therapeutic drug monitoring may be useful in monitoring patients switching from originator infliximab to biosimilar infliximab [62].

A Danish real-world analysis (DANBIO registry) in which patients with inflammatory diseases were switched from reference infliximab to biosimilar CT-P13 found no difference of serum drug concentration or presence of anti-drug antibodies (ADAs) after versus before the switch [63]. An 11-month analysis of registry data on 768 patients with RA, psoriatic arthritis (PsA), and axial spondyloarthritis (AxSpA) who were switched from originator infliximab to CT-P13 showed no impact of the switch on disease activity. However, 15% of patients stopped treatment after the switch, which warrants further investigation but may be related to a possible “nocebo” effect—that is, a phenomenon in which patients who are aware that they are being switched to a drug that they believe is less effective may exhibit a worse response compared with the possible outcome if they were not aware of the switch [63, 64]. Apart from the nocebo effect, loss of efficacy often associated with continued biologic treatment may be unfairly attributed to the biosimilar. Both these reasons may lead to patients stopping a biosimilar after unblinded versus blinded switch [63].

Communication with patients or lack thereof may contribute to biosimilar discontinuation. A study by Tweehuysen and colleagues [64], looking at two biosimilar transition projects (CT-P13 and SB4) in patients with inflammatory diseases, demonstrated that an enhanced communication strategy resulted in higher acceptance and persistence rates with biosimilars.

While some data are available, more are required to ensure the long-term safety and efficacy of these drugs in a real-world setting in order to give rheumatologists the confidence to use these medicines in their practice. Real-world data in the Middle East region are lacking and further work needs to be carried out to ensure physicians are confident to prescribe biosimilars.

#### Automatic substitution and interchangeability

Some physicians have expressed concerns regarding automatic substitution at the pharmacy level [21]. Automatic substitution allows a pharmacist to switch a reference biologic to a biosimilar without the approval or knowledge of the prescribing physician [43]. A survey of US, Latin American, and European physicians showed that physicians believe it is imperative that they retain the authority to choose the biosimilars for their patients. Automatic substitution of biosimilars is not recommended in clinical practice by most rheumatology societies of various countries, and prescribing decisions should be assigned to the physician with patient consent [27].

In the USA, only with an FDA-approved interchangeability designation would automatic substitution be allowed at the pharmacy. For a biosimilar to be considered interchangeable, it must have data demonstrating that switching does not incur safety risks [27]. Currently, no biosimilars meet the criteria for the FDA definition of interchangeability.

The EMA does not provide a definition of interchangeability but leaves the decision to its member countries, and most of these countries do not allow automatic substitution [27].

### Position statements: key messages

Many rheumatology societies have issued position statements on biosimilars to aid in clinical decision-making. These are summarized in the Online resource (Table 4).

A common important theme between all the position statements is that any switching decision between biosimilars and the original product must be the decision of the attending physician and performed only with informed approval from the patient.

Currently, there is scant guidance regarding approval, pharmacovigilance, and use of biosimilars in the Middle East region, particularly considering the availability of intended copies in the region. Appropriate recommendations and guidance, as well as robust real-world data, are required to ensure safe and effective use of these medicines in clinical practice. These will help ensure physicians have confidence to prescribe biosimilars and help ensure their ongoing pharmacovigilance.

## Recommendations for the use of biosimilars in the Middle East

The findings of the above integrative review, as well as the authors’ experiences with using biosimilars, were used as the basis of a set of recommendations for these drugs proposed by the authors.

The authors’ proposed recommendations on the use of biosimilar drugs in patients with RA are as follows:

- There is a need for national databases or registries to record details of biologic switching, particularly adverse event profiles, so as to obtain safety data postswitching.
- The less expensive drug is a reasonable first therapeutic choice in naive patients refractory to conventional DMARDs.
- Clinical and real-world data on biosimilars must be considered before deciding to switch patients to biosimilar drugs. Postmarketing surveillance is important to collect this real-world data.
- Switching should remain a case-by-case clinical decision made jointly by the physician and patient, with a minor role played by other parties including insurance companies and health authorities, and supported by scientific evidence. Dialog with the patient is vital when making the switching decision and patients should approve the switch to a biosimilar medicine.
- When considering switching treatment, it is important that the switches are recorded appropriately in the patient’s medical history, including the brand name and batch number of the biosimilar product [46].
- Automatic substitution should not take place at the pharmacy level.
- A true biosimilar agent, approved by the EMA or FDA, could be regarded as trustworthy and must be differentiated from intended copies, which are available in some Middle Eastern markets without stringent biosimilarity studies.
- There is a need for enhanced training of regulatory authorities on how to evaluate biosimilars and differentiate these from intended copies.
- Each Middle Eastern country should develop a working group with an interest in biosimilars who can review current drugs on their healthcare market and provide appropriate prescription advice to other rheumatologists.
- Pharmacovigilance processes and traceability of biosimilars must be improved in the Middle East. Biologic and biosimilar medicines must be referred to by their brand name and batch number rather than their INN, to help enable better traceability.

These recommendations have some limitations, however. Firstly, although the literature search aimed to be thorough, some country-specific guidance may only be available locally. Additionally, because of the heterogeneous nature of healthcare in the Middle East, it may be challenging to implement these guidelines consistently across the region.

## Conclusion

The use of biosimilar medicines in the Middle East provides an important opportunity to treat more rheumatology patients with biologic drugs. However, in order to be able to prescribe these medicines, physicians must feel confident that these medicines are safe and effective for their patients. This will require more robust real-world experience with biosimilar medicines in the Middle East region, particularly with regard to long-term data and switching.

Any decision to switch should be made on a case-by-case basis supported by scientific evidence and must be patient, disease, and product specific—originator biologics and biosimilars are not allowed to be automatically substituted without physician involvement or patient awareness.

Additionally, it is important for both regulators and physicians to be aware fully of the differences between legitimate biosimilar medicines and intended copies. The recommendations outlined in this paper may provide a basis for regulator and physician decision-making with regard to biosimilar medicines used for rheumatic diseases in the Middle East region.

## Electronic supplementary material

ESM 1 (DOCX 78 kb)
