# Supplementary material for: Biosimilars in rheumatology: recommendations for regulation and use in Middle Eastern countries
Source: Clin Rheumatol. 2018 Feb 6;37(5):1143–52. doi: 10.1007/s10067-018-3982-9 (PMC5913383; doi:10.1007/s10067-018-3982-9)
Supplement: Supplementary file 1 — (DOCX 78 kb) [file 10067_2018_3982_MOESM1_ESM.docx]

# Online resources

## Table 1: Biosimilars products and guidelines available in countries of the Middle East

| Country  Regulatory Authority | Biosimilar Regulation | Biosimilars approved for rheumatology conditions | Intended copies available in the region |
| --- | --- | --- | --- |
| Bahrain  No authority identified | No guidelines found |  |  |
| Iraq  Ministry of Health | Draft guidance in development |  |  |
| Iran  Iranian National Regulatory Authority | No guidelines found | None – copy biologics only (known locally as ‘biogenerics’) [1] | Zytux (rituximab)  Altebrel (etanercept) |
| Jordan  Jordan Food and Drug Administration | [Guideline](https://bricwallblog.com/2015/10/05/jordan-fda-issues-a-guideline-for-the-registration-of-biosimilars/) for the registration of biosimilars [2] | CT-P13 (biosimilar infliximab) |  |
| Kuwait  Drug and Food Control Administration | No guidelines found |  |  |
| Lebanon  Lebanese Ministry of Public Health (MOPH) [3] | Guidance and List of Requirements for Registration of Biosimilars Products [4] | FDA-approved biosimilars for infliximab, etanercept, and adalimumab |  |
| Oman  Ministry of Health | No guidelines found |  |  |
| Palestine  Ministry of Health | No guidelines found |  |  |
| Qatar  Pharmacy and Drug Control Department | No guidelines found | None available |  |
| Saudi Arabia  Saudi Food and Drug Authority | Guidelines on Biosimilar Products [5] | CT-P13 (for Infliximab) [6] |  |
| Syria  No authority identified | No guidelines found |  |  |
| United Arab Emirates | Draft guidance: Abuelkhair M., Abdu S., Godman B. and et al, "Imperative to consider multiple initiatives to maximize prescribing efficiency from generic availability: case history from Abu Dhabi." *Expert Rev Pharmacoecon Outcomes Res.,* 2012. | Nothing approved to date | Nothing approved |
| Yemen  Supreme Board for Drugs and Medical Appliances | No guidelines found |  |  |

1. Quintiles (2016) Middle East and North Africa. Biosimilars Knowledge Connect.  [http://www.biosimilarsknowledgeconnect.com/biosimilars-by-region/middle-east-and-north-africa](http://www.quintiles.com/microsites/biosimilars-knowledge-connect/biosimilars-by-region/middle-east-and-north-africa%20Last%20accessed%20July%202017) Accessed July 2017
2. BRIC Wall (2015) Jordan FDA issues a guideline for the registration of biosimilars. <https://bricwallblog.wordpress.com/2015/10/05/jordan-fda-issues-a-guideline-for-the-registration-of-biosimilars/> Accessed November 2017
3. Farhat F et al (2016) Review and results of a survey about biosimilars prescription and challenges in the Middle East and North Africa region. SpringerPlus 5:2113. <https://doi.org/10.1186/s40064-016-3779-8>.
4. Republic of Lebanon (2016) Ministry of Public Health. Guidance for Registration of Similar Biological Medicinal products (Biosimilars). <http://www.moph.gov.lb/en/view/6917/guidance-and-list-of-requirements-for-registration-of-biosimilars-products> Accessed November 2017
5. Saudi Food and Drug Authority (2017) Guideline on Biosimilar Products: quality considerations. <https://www.sfda.gov.sa/ar/drug/resources/DocLib2/Guideline-on-biosimilar-products.pdf> Accessed November 2017
6. Alhomaidan AM et al (2016) Pricing of biosimilars in Saudi Arabia*.* Generics and Biosimilars Initiative Journal (GaBI Journal). 5(1):27–29. <https://doi.org/10.5639/gabij.2016.0501.007>.

## 2. Table 2: Copy biologics for inflammatory diseases [1–3]

Copies of biologics currently available for the treatment of inflammatory diseases for which data has been published in peer-reviewed journals or presented at intentional scientific meetings.

| **Reference Product** | **Copy biologic** | **Brand name** | **Authorised by EMA** | **Authorised by FDA** |
| --- | --- | --- | --- | --- |
| **Adalimumab**  **Humira (AbbVie) EMA authorisation: 2003** | ABP 501 | Amgevita (Amgen) | 2017 | 2017 |
|  | BI 695501 | Cyltezo (Boehringer-Ingelheim) |  | 2017 |
|  | CHS-1420 | (Coherus BioSciences) |  |  |
|  | GP-2017 | (Sandoz) |  |  |
|  | M923 | (Momenta) |  |  |
|  | SB5 | Imraldi (Samsung Bioepsis) | 2017 |  |
|  | ZRC-3197† | Exemptia (Zydus Cadila) |  |  |
|  | PF-06410293 | (Pfizer) |  |  |
| **Etanercept**  **Enbrel (Pfizer) EMA authorisation: 2000** | CHS-0214 | (Coherus BioSciences) |  |  |
|  | GP-2015 | Erelzi (Sandoz) | 2017 | 2016 |
|  | HD203* | Davictrel (Hanwha/Merck) |  |  |
|  | SB4 | Benepali (Samsung Bioepsis) | 2016 |  |
| **Infliximab**  **Remicade (Janssen) EMA authorisation: 1999** | BOW015† | Infimab (Ranbaxy) |  |  |
|  | CT-P13 | Remsima (Celltrion) | 2013 | N/A |
|  |  | Inflectra (Pfizer) |  | 2016 |
|  | PF-06438179 | (Pfizer) |  |  |
|  | SB2 | Flixabi (Samsung Biopsis) | 2016 | N/A |
|  |  | Renflexis (Samsung Biopsis) | N/A | 2017 |
|  | ABP 710 | (Amgen) |  |  |
|  | LBEC0101 | (LG Chem) |  |  |
|  | ENIA11 | TuNEX (Mycenax) |  |  |
| **Rituximab**  **MabThera (Roche) EMA authorisation: 2008** | CT-P10 | Truxima (Teva/Celltrion) | 2017 |  |
|  | GP-2013 | (Sandoz) |  |  |
|  | PF-05280586 | (Pfizer) |  |  |
|  | ABP 798 | (Amgen) |  |  |
|  | BI69550 | (Boehringer Ingelheim) |  |  |
| *** Approved in Korea**  **† Approved in India** | | | | |

1. Dorner T, Strand V, Cornes P et al (2016) The changing landscape of biosimilars in rheumatology. Annals of the Rheumatic Diseases 75:974–982. <https://doi.org/10.1136/annrheumdis-2016-209166>.
2. Kanters TA, Stevanovic J, Huys I et al (2017) Adoption of Biosimilar Infliximab for Rheumatoid Arthritis, Ankylosing Spondylitis, and Inflammatory Bowel Diseases in the EU5: A Budget Impact Analysis Using a Delphi Panel. Frontiers in Pharmacology 8:322. <https://doi.org/10.3389/fphar.2017.00322>.
3. Moots R, Azevedo V, Coindreau JL et al (2017) Switching Between Reference Biologics and Biosimilars for the Treatment of Rheumatology, Gastroenterology, and Dermatology Inflammatory Conditions: Considerations for the Clinician. Current Rheumatology Reports 19(6):37. <https://doi.org/10.1007/s11926-017-0658-4>.

## 3. Literature search

Searches were performed using a string of terms:

• American College of Rheumatology (ACR) abstracts: biosimilars

• ACR abstracts: biosimilar

• European League Against Rheumatism (EULAR) abstracts: biosimilars

• EULAR abstracts: biosimilar

• Pubmed: Approval, guidelines, biosimilars, rheumatology

• Pubmed: Position, biosimilars, rheumatology

• Pubmed: Switch, biosimilars, rheumatology

• Pubmed: Regulation, biosimilar, rheumatology

• Pubmed: Intended copy, rheumatology

• Plus manual search for position statements and regulatory guidelines

## Table 3: Clinical and real life data from literature analysis

| First Author | No of patients | Study design | Intervention | Follow up after starting biosimilar | Results | Limitations |
| --- | --- | --- | --- | --- | --- | --- |
| Clinical Studies | | | | | |  |
| Adalimumab | | | | | |  |
| Alten R et al 2017 [1] | 730  RA | RCT, double blind | 1:1 FKB327  Reference adalimumab | 24 wk | - Equivalent efficacy - Comparable safety profiles, anti-drug antibody formation and PK |  |
| Cohen SB et al 2015, 2016a & 2016b [2,3,4] | 526  RA | RCT, double blind followed by open label phase | 1:1 ABP 501  Reference adalimumab | 26 wk (DB)  72 wk (OL) | - Double blind: equivalent clinical efficacy and safety - Efficacy was maintained in the long-term extension phase - Safety and efficacy was similar between subjects who transitioned from reference adalimumab and those who continued on ABP 501 | Necessary data to compare switched and non-switched patients are not available [5] |
| Cohen S et al 2017 [6] | 645  RA | RCT, double-blind | 1:1  BI 695501  Reference adalimumab (USA) | 24 wk | - Similar efficacy, safety and immunogenicity |  |
| Eremeeva A et al 2016 [7] | 94  Healthy | PK equivalence study  RCT, single-blind | 1:1  BCD-057  Reference adalimumab | 2 mo | - Equivalent PK, safety and immunogenicity | Short term study in healthy population [7] |
| Hillson J et al 2016 [8,9] | 324  Healthy | RCT  Immunogenicity study | 1:1:1  M923  Adalimumab (US)  Adalimumab (UK) | 10 wk | - Equivalent PK and comparable immunogenicity with M923 vs. US- and EU-sourced reference adalimumab | Short term study in healthy population [8,9] |
| Kaur P et al 2017 [10] | 203  Healthy | PK equivalence study  RCT, single-blind | 1:1:1  ABP 501  Adalimumab (EU)  Adalimumab (USA) | n/a  (single application) | - Similar PK profiles for all three products - Similar safety and tolerability of all three products - Similar anti-drug antibody rates for all three groups | Short term study in healthy population [10] |
| Weinblatt ME et al 2015 & 2016 [11,12] | 544  RA | RCT, double-blind | 1:1  SB5  Reference adalimumab | 52 wk | - Equivalent efficacy and similar safety profile, PK, and immunogenicity to adalimumab |  |
| Wynne C et al 2015 [13] | 327  Healthy | PK equivalence study  RCT, double-blind | 1:1:1  BI 695501  Reference adalimumab (USA)  Reference adalimumab (EU) | 2 mo | - Bioequivalent - Similar safety and tolerability profiles | Short term study in healthy population [13] |
| Etanercept | | | | | |  |
| Afonso M et al 2016 [14] | 51  Healthy | PK equivalence study  RCT, open label, cross over | GP2015 AI or PFS  Second subcutaneous injection of GP2015 via PFS or AI vice versa | 28 d | - Bioequivalent PK of GP2015 administered by AI or PFS - GP2015 was well tolerated (safety profile consistent with previous reports for reference etanercept) | Data from the following outcomes was not assessed: Pre- and post-switch PK, safety, or immunogenicity evaluations [5] |
| Bae S et al 2014 [15] | 294  RA | RCT, double blind | 1:1 HD203  Reference etanercept | 48 wk | - Equivalence efficacy with reference etanercept - Comparable safety profile |  |
| Emery P et al 2016 [16,17] | 245  RA | Open label (Czech & Poland) of Phase III RCT, double blind | Continued  Reference etanercept or switched to SB4 | 2 yr | - SB4 — well tolerated and effective over two years - Efficacy, safety, and immunogenicity were comparable between the maintenance and switch patients | Immunogenicity was assessed before and after the first dose, but not after the transition.  The effect of the switch on PK parameters was not assessed [5] |
| Kivitz AJ et al 2016 [18] | 521  PsA & PsO | RCT | CHS-0214  Reference etanercept | 12 wk | - Similar efficacy and safety in both groups | Not a switch study [18] |
| Lee Y et al 2015 [19] | 138  Healthy | PK equivalence study  RCT, double-blind | 1:1:1  SB4  Reference etanercept (USA)  Reference etanercept (EU) | 28 d | - Similar PK profiles for all three - Similar safety and tolerability for all three | Short term study in healthy population [19] |
| Vencovsky J et al 2015 [20,21] | 596  RA | RCT, double blind | 1:1  SB4  Reference etanercept | 52 wk | - Comparable efficacy and safety Immunogenicity profile was lower in SB4 compared to reference etanercept | Immunogenicity was assessed before and after the first dose, but not after the transition.  The effect of the switch on PK parameters was not assessed [5] |
| Vencovsky J et al 2017 [22] | 596  RA | RCT, double-blind | 1:1  SB4  Reference etanercept | 24 wk | - Significantly fewer patients takingSB4 developed anti-drug antibodies or experienced injection site reactions compared to reference etanercept | Immunogenicity was assessed before and after the first dose, but not after the transition.  The effect of the switch on PK parameters was not assessed [5] |
| Von Richter et al 2017 [23] | 54  Healthy | PK equivalence study  RCT, crossover | 1:1  GP2015  Reference etanercept | 29 d | - Similar PK - Safety profile consistent with previous reports of reference etanercept | Carried out only in healthy subjects [23] |
| Infliximab | | | | | |  |
| Choe J et al 2016 [24,25,26] | 583  RA | RCT, double blind | 1:1  SB2  Reference infliximab | 54 wk | - Comparable long-term efficacy, safety and immunogenicity | Not a switch study [5,24,25,26] |
| Denisov L et al 2017 [27] | 484  AS | Comparative study – comparison of 3 RCTs (all double blind) | BCD-055  Reference infliximab | 54 wk | - Similar efficacy, safety and PK | Not a switch study [27] |
| Jørgensen KK et al  NOR-SWITCH [28] | 481  Inflammatory diseases | RCT, double-blind, non-inferiority trial | Continue after reference infliximab treatment for 6 mo vs switch to CT-P13 | 52 wk | - Similar efficacy, safety and immunogenicity observed in both arms | 1.Multiple switch effects not considered  2. The results were cross-indication because the study was not powered for individual diseases [28, 29] |
| Kaur P et al 2016 [30] | 99  Healthy | PK equivalence study  RCT, Single-blind | 1:1  ABP 710  Infliximab | n/a  (single application) | - Similar PK profiles for both - Similar safety and tolerability for both - Similar anti-drug antibody rates for both | Short term study in healthy population [30] |
| Kay J et al 2014 [31, 32] | 189  RA | RCT, double blind phase followed by open label (after wk 22)  OL: IFX responders switched to BOW015 | 2:1  BOW015  IFX | 46 wk | - BOW015 – well tolerated with similar ACR response - Similar immunogenicity in double blind and open label phase - Bioequivalent |  |
| Park SH et al 2015 [33] | 142  Healthy | PK equivalence study  RCT, Single-blind, cross over | 1:1:1  Reference infliximab (USA)  Reference infliximab (EU)  CTP-13 | 57 d | - Similar PK profiles for all three - Similar tolerability for all three | Doses administered & PK assessments taken at different time points in this population vs. AS population |
| Park W et al 2013 & 2017  PLANETAS [34,35] | 250  AS | Phase I, RCT, double blind followed by open label extension study | 1:1  Reference infliximab  CTP-13 | 2 yr | - Equivalent PK - Comparable efficacy and safety profile | This study was not designed to evaluate  the non-inferiority or equivalence of switching from reference IFX to  CT-P13 vs continued treatment with CT-P13 [35] |
| Shin D et al 2015 [36] | 159  Healthy | PK equivalence study  RCT, single-blind | 1:1:1  SB2  Reference infliximab | 71 d | - Equivalent PK - Comparable efficacy and safety profile | Short term study in healthy population [36] |
| Smolen J et al 2016a [37] | 396  RA | Cross over period analysis  RCT, double blind | 1:1  SB2  Reference infliximab (USA)  Reference infliximab (EU) | 78 wk | - Comparable tolerability, immunogenicity and efficacy between the INF/SB2, INF/INF, and SB2/SB2 transitions | More data and follow-up required (pharmacovigilance, registry data and/or additional studies) to demonstrate the safety of switching [5] |
| Tanaka Y et al 2017 [38] | 71  RA | Open label  Patients completed 54 wk of treatment in a phase I/II study (PI/II) received CTP13 | CTP-13 | 134 wk | - CT-P13 was well tolerated in maintenance & switch patients | Sample size was set to analyze the primary endpoint for phase I/II study but not the extension study findings [38] |
| Udata C et al 2016 [39] | 151  Healthy | PK equivalence study  RCT, double blind | 1:1  PF-06438179  Reference infliximab (USA)  Reference infliximab (EU) | 12 wk | - Comparable immunogenicity across all three arms - PK similarity and comparable safety and immunogenicity profiles to reference infliximab | Needs confirmation in patients with RA |
| Yoo DH et al 2013 & 2017 [40.41]  PLANETRA | 606  RA | RCT, double blind followed by open label extension study | 1:1  CTP-13 Reference infliximab | 2 yr | - Equivalent efficacy - Comparable PK profile and immunogenicity - Comparable safety profile | Because this is a post-hoc analysis, the comparable efficacy data should be interpreted with care [41]  Limited evaluation time points for ACR20 response [41] |
| Yoo DH et al 2014 [42] | 213  Healthy | PK equivalence study  RCT, double-blind | 1:1:1  CT-P13  Reference infliximab (USA)  Reference infliximab (EU) | 8 wk | - Equivalent PK profiles for all groups | Healthy subjects |
| Becker JP et al 2014 [43] | 220  RA | PK equivalence study  RCT, double blind, crossover | 1:1:1  PF-05280586 Rituximab (US)  Rituximab (EU) | 17 wk | - Similar PK - Improvement in clinical score - All three treatments were generally well tolerated | Short term data [43] |
| Eremeeva A et al 2016b [44] | 160  RA | RCT, double-blind | 1:1  BCD-020  Reference rituximab | 6 mo | - Equivalent efficacy | Long-term efficacy and safety needs to be evaluated [44] |
| Nasonov E et al 2016 [45] | 160  RA | RCT, double blind crossover | 1:1  BCD-020  Reference rituximab | 1 yr | - Similar efficacy, safety and immunogenicity - Switching did not affect patient outcomes |  |
| Smolen J et al 2016b [46] | 325  RA | PK equivalence study  RCT, double-blind | 1:1  GP2013  Reference rituximab | 52 wk | - Equivalent PK - Comparable efficacy and safety profile |  |
| Suh CH et al 2016 [47] | 189  RA | PK equivalence study  RCT | 1:1:1  CT-P10  Reference rituximab (USA)  Reference rituximab (EU) | 24 wk | - PK equivalence demonstrated - Comparable safety profiles |  |
| Suh C et al 2017 [48] | 372  RA | RCT, double blind | 1:1:1  CT-P10  Reference rituximab (USA)  Reference rituximab(EU) | 48 wk | - Comparable efficacy, pharmacodynamics, immunogenicity and safety for one year |  |
| Yin D et al 2014 [49] | 220  RA | PK equivalence study  RCT, double-blind | 1:1:1  PF-05280586  Reference rituximab (USA)  Reference rituximab (EU) | 2 wk | - Similar PK in all 3 arms - All arms well tolerated | No switch study yet |
| Yoo DH et al 2016 [50,51,52] | 154  RA | RCT, double blind  Open label extension (87 patients) | 1:1  CT-P10  Reference rituximab | 72 wk (DB)  2 yr (OL) | - Comparable efficacy and safety in switch vs. maintained patients on CT-P10 | Different efficacy measures were reported for the double blind and open label phases so efficacy pre- & post-switch could not be compared  Immunogenicity was only assessed pre-switch [5] |
| Real life data | | | | | |  |
| Adalimumab | | | | | |  |
| Agarwal M et al 2017 [53] | 29  JIA + uveitis | Patient data – retrospective review of data | ZRC 3197 |  | - Safe and rapidly effective agent - No comparison with reference adalimumab was made | Small sample size [53] |
| Bandyopadhyay S et al 2017 [54] | 52  AS | Patient data – retrospective | Adalimumab biosimilar – not specified | 48 wk | - Adalimumab was effective in treating AS patients - Stable disease with no worsening after stopping treatment for 6 months | Small sample size [54] |
| Kaushik V et al 2017 [55] | 200  RA + AS | Retrospective analysis of patient data | Adalimumab biosimilar (Cadila Healthcare Ltd., India) | 6 mo | - Similar safety profile to reference adalimumab (comparison with adalimumab clinical trials) | Retrospective, not an RCT and small sample size [55] |
| Etanercept | | | | | |  |
| Emery P et al 2017 [56] | 147  Inflammatory Arthritis | Patient data review after switch from etanercept  Swedish Rheumatology Quality Register (SRQ) | SB4 | 22 wk | - Switch was acceptable to most patient - Low mean disease activity maintained | Short term outcomes data [56] |
| Holroyd C et al 2017 [57] | 92  RA + PsA + AS | Retrospective comparison cross over observation | Reference etanercept to SB4 switch patients in the SMaRT study | 6 mo | - Low rate of discontinuations due to lack of efficacy or adverse events | Small number of patients, single-centre study with no comparator arm [5] |
| Shivpuri A et al 2016 [58] | 69  JIA | Retrospective patient data review (India) | Etanercept biosimilars (Cipla & Intas) | 6 mo | - Cipla & Intas licensed were safe & effective - Comparable to reference etanercept | Small number of patients; single-centre study with no comparator arm and short follow up [58] |
| Infliximab | | | | | |  |
| Abdalla A et al 2017  UK [59] | 34  Inflammatory arthritis | Patient data | CT-P13 | 15.8 mo | - Similar efficacy and safety | 1. Small sample size,  2. Single center  3. Observational (not RCT)  4.Lack of data on menopausal  status of the women  5. Lack of serum  immunogenicity markers before and after switch [60] |
| Agrawal I et al 2017 [61] | 68  AS | Retrospective – data obtained from Indian centres | BOW015 | 6 mo | - Significant improvement in disease scores - No comparison to IFX was made | Small sample size; short follow up [61] |
| Akrout W et al 2017 [62] | 99  RA + SpA + PsA | Retrospective – data of patients who switched from IFX | Infliximab biosimilar – not specified | 6 mo | - Up to 23% transition failure rate – no clear reason given (but authors note that it is difficult to assess the inefficacy of the transition to biosimilar infliximab only according to patient complaints) | Short term; inefficacy or loss of efficacy was based on patient complaint [62] |
| Avouac J et al 2017 [63] | 260  Rheumatic disease | Retrospective – patient data from one hospital in France | Infliximab biosimilar – not specified | 34 wk | - No changes in efficacy and safety - 26% of patients discontinued infliximab biosimilar, mainly those with SpA due to a subjective increase in disease scores | Subjective outcome [63] |
| Batticciotto A et al 2016  Italy [64] | 31  SpA | Patient data | CT-P13 | 6 mo | - No differences in efficacy or tolerability profile was observed 6 months after switch to biosimilar | Short term data [64] |
| Codreanu C et al 2016 [65] | 151  RA + AS | Multi-centre, non-interventional, observational study in Romania, Czech Republic and Bulgaria | CT-P13 | 4 mo | - CT-P13 shown to be safe and effective in a real-life setting | Short term data [65] |
| Glintborg B et al 2016  DANBIO registry [66] | 768  Inflammatory Arthritis | Retrospective – data obtained from Danish Rheumatologic Biobank and the DANBIO Registry | Reference infliximab patients switched to CT-P13 | 11 mo | - Disease activity was unaffected after switch - Similar fluctuations before and after switch - 15% stopped treatment after switch | Database/registry analyses – not RCT [5] |
| Glintborg B et al 2016  DANBIO registry [67,68] | 647  Inflammatory Arthritis | Retrospective – data obtained from DANBIO Registry | Reference infliximab patients switched to CT-P13 | 3 mo | - Disease activity was unaffected after non-medical switch - ~6% stopped treatment due to lack of effect or adverse event - No impact on serum drug concentration or presence of anti-drug antibodies | Database/registry analyses – not RCT [5]; short term data [67,68] |
| Joseph J et al 2017  Cyprus [69] | 160  Rheumatologic disease | Retrospective recording of efficacy & safety of biosimilar infliximab CT-P13. Survey of rheumatologists | N/A | ≥3 m-<1 yr | - 30% discontinued (most within 6 mo of starting drug) - Numbers remaining on the drug and adverse effects were similar to previous large studies of infliximab - Rheumatologists felt that therapeutic target has been met in the majority of cases | Survey / retrospective rather than an RCT [69] |
| Presberg Y et al 2017 [70] | 89  Rheumatic disease | Patient data review | Switched from reference infliximab to CT-P13 | 10 mo | - No new safety or efficacy concerns reported | Small sample size; data review rather than clinical trial [70] |
| Rubio E et al 2016 [71] | 78  Rheumatic disease | Patient follow up study  Visits every 3 mo | CT-P13 | 8.3 mo | - Safety data are similar to those shown in PLANETRAS and PLANETAS, both in biologic naive & switch patients | Short follow up period [71] |
| Sokka T et al 2015 [72] | 39  RA /SpA | Patient data review | Biosimilar infliximab – not specified | 11 mo | - No new safety signals observed in switch patients | Small sample size and short follow up [72] |
| Sung YK et al 2017 [73] | 98  RA | Patient data review from BIOlogics Pharmacoepidemiologic StudY (BIOPSY) | Infliximab or biosimilar infliximab patients | 9 mo | - Comparable drug persistency | Small sample size and short follow up [73] |
| Tweehuysen L et al 2016 [74] | 75  RA/SpA/PsA | Observational – patient data review | Biosimilar infliximab – not specified | 6 mo | - Efficacy, safety and immunogenicity not affected over 6 mo - 23% of the patients discontinued biosimilar IFX due to adverse events or nocebo | Small sample size and short follow up [74] |
| Yazici Y et al 2016 [75] | 3,018  RA | Turkish Database:  Retrospective comparative – patient data | Continued on reference infliximab, switched from infliximab to CT-P13 | 12 mo | - High percentage (82%) of CT-P13 switch patients discontinued & most returned to reference infliximab | Study not powered to investigate reasons for discontinuations |
| Rituximab | | | | | | |
| Roshique KK & Ravindran V [76] | 21 RA | Prospective observational study | Switched to intended copy rituximab (Reditux) |  |  |  |

**AI, Autoinjector; AS, ankylosing spondylitis; IFX, infliximabPFS, Pre-filled syringes; PK, Pharmacokinetic; PsA, Psoriatic arthritis; PsO, chronic plaque psoriasis; RA, rheumatoid arthritis; RCT, Randomized controlled trial; SpA, Spondyloarthritis**

1. Alten R, Glover J, Matsunaga N et al (2017) OP0021 Efficacy and safety results of a phase iii study comparing fkb327, an adalimumab biosimilar, with the adalimumab reference product in patients with active rheumatoid arthritis. Annals of the Rheumatic Diseases 76:59. <https://doi.org/10.1136/annrheumdis-2017-eular.2220>.
2. Cohen SB, Genovese MC, Choy EH et al (2015) Randomized, Double-Blind, Phase 3 Study of Efficacy and Safety of ABP 501 Compared with Adalimumab in Subjects with Moderate to Severe Rheumatoid Arthritis [abstract]. Arthritis & Rheumatology 2015;67(suppl 10). <http://acrabstracts.org/abstract/randomized-double-blind-phase-3-study-of-efficacy-and-safety-of-abp-501-compared-with-adalimumab-in-subjects-with-moderate-to-severe-rheumatoid-arthritis/> Accessed January 2018
3. Cohen S, Zhang N, Kaur P (2016) FRI0191 Biosimilar Candidate ABP 501: Additional Efficacy Analyses from The Phase 3 Study. Annals of the Rheumatic Diseases 75:499. <https://doi.org/10.1136/annrheumdis-2016-eular.3734>.
4. Cohen S, Pablos JL, Zhang N et al (2016) ABP 501 Long-Term Safety/Efficacy: Interim Results from an Open-Label Extension Study [abstract]. Arthritis & Rheumatology 68(suppl 10). <http://acrabstracts.org/abstract/abp-501-long-term-safetyefficacy-interim-results-from-an-open-label-extension-study/>. Accessed July 2017.
5. Moots R, Azevedo V, Coindreau JL et al (2017) Switching Between Reference Biologics and Biosimilars for the Treatment of Rheumatology, Gastroenterology, and Dermatology Inflammatory Conditions: Considerations for the Clinician. Current Rheumatology Reports 19(6):37. <https://doi.org/10.1007/s11926-017-0658-4>.
6. Cohen S, Alonso-Ruiz A, Klimiuk P et al (2017) FRI0189 Similar efficacy and safety of biosimilar candidate BI 695501 and adalimumab originator reference product in patients with moderate to severe active rheumatoid arthritis: 24 week results from a phase III clinical study (voltaire®-ra). Annals of the Rheumatic Diseases 76:553. <https://doi.org/10.1136/annrheumdis-2017-eular.3405>
7. Eremeeva A, Fogt S, Chernyaeva E et al (2016) AB0034 Pharmacokinetics and Safety of BCD-057, Adalimumab Biosimilar Candidate, Compared To Humira in Healthy Volunteers (Results of Phase I Clinical Study). Annals of the Rheumatic Diseases 75:908. <https://doi.org/10.1136/annrheumdis-2016-eular.2114>.
8. Hillson J, Mant T, Ganguly T et al (2016) Assessment of Comparative Immunogenicity in Biosimilar Development: Immunogenicity and Pharmacokinetics Following a Single Dose of M923, a Proposed Biosimilar for Reference Adalimumab (HUMIRA®), Compared with US- and EU-Sourced Reference Adalimumab in Healthy Subjects [abstract]. Arthritis & Rheumatology 68(suppl 10). <http://acrabstracts.org/abstract/assessment-of-comparative-immunogenicity-in-biosimilar-development-immunogenicity-and-pharmacokinetics-following-a-single-dose-of-m923-a-proposed-biosimilar-for-reference-adalimumab-humira/>. Accessed July 2017.
9. Hillson J, Mant T, Ganguly T et al (2016) FRI0182 A Single Dose Study Comparing Pharmacokinetics, Safety, and Immunogenicity of M923 (A Proposed Biosimilar To Adalimumab), US-Sourced Adalimumab, and EU-Sourced Adalimumab in Healthy Subjects. Annals of the Rheumatic Diseases 75:495–496. <https://doi.org/10.1136/annrheumdis-2016-eular.3706>.
10. Kaur P, Chow V, Zhang N et al (2017) A randomised, single-blind, single-dose, three-arm, parallel-group study in healthy subjects to demonstrate pharmacokinetic equivalence of ABP 501 and adalimumab. Annals of the Rheumatic Diseases 76:526–533. <https://doi.org/10.1136/annrheumdis-2014-eular.2166>.
11. Weinblatt ME, Baranauskaite A, Niebrzydowski J et al (2015) A Phase III, Randomized, Double-Blind Clinical Study Comparing SB5, an Adalimumab Biosimilar, with Adalimumab Reference Product (Humira®) in Patients with Moderate to Severe Rheumatoid Arthritis Despite Methotrexate Therapy (24-week Results) [abstract]. Arthritis & Rheumatology 67(suppl 10). <http://acrabstracts.org/abstract/a-phase-iii-randomized-double-blind-clinical-study-comparing-sb5-an-adalimumab-biosimilar-with-adalimumab-reference-product-humira-in-patients-with-moderate-to-severe-rheumatoid-arthritis/>. Accessed July 2017
12. Weinblatt M, Baranauskaite A, Niebrzydowski J et al (2016) Sustained Efficacy and Comparable Safety and Immunogenicity after Transition to SB5 (an Adalimumab Biosimilar) Vs. Continuation of SB5 or Reference Adalimumab (Humira®) in Patients with Rheumatoid Arthritis: Results of Phase III Study [abstract]. Arthritis & Rheumatology 68(suppl 10). <http://acrabstracts.org/abstract/sustained-efficacy-and-comparable-safety-and-immunogenicity-after-transition-to-sb5-an-adalimumab-biosimilar-vs-continuation-of-sb5-or-reference-adalimumab-humira-in-patients-with-rheumatoi/>. Accessed July 2017.
13. Wynne C, Petkova M, Rombout F et al (2015) BI 695501, a Proposed Biosimilar for Adalimumab, Shows Bioequivalence to Adalimumab Reference Products in a Randomized, Double-Blind Phase I Trial in Healthy Subjects [abstract]. Arthritis & Rheumatology 67(suppl 10). <http://acrabstracts.org/abstract/bi-695501-a-proposed-biosimilar-for-adalimumab-shows-bioequivalence-to-adalimumab-reference-products-in-a-randomized-double-blind-phase-i-trial-in-healthy-subjects/> Accessed July 2017.
14. Afonso M, Kollins D, Macke L et al (2016) THU0144 Pharmacokinetics and Safety of GP2015, A Proposed Etanercept Biosimilar, Administered Subcutaneously by An Autoinjector or Prefilled Syringe in Healthy Male Subjects. Annals of the Rheumatic Diseases 75:233–234. <https://doi.org/10.1136/annrheumdis-2016-eular.1304> Accessed July 2017
15. Bae SC et al (2014) A Randomized, Double-Blind, Phase 3 Equivalence Trial Comparing the Etanercept Biosimilar, HD203, with Etanercept (Enbrel®), in Combination with Methotrexate (MTX) in Patients with Rheumatoid Arthritis (RA). Arthritis & Rheumatology 66(suppl 10). <http://acrabstracts.org/abstract/a-randomized-double-blind-phase-3-equivalence-trial-comparing-the-etanercept-biosimilar-hd203-with-etanercept-enbrel-in-combination-with-methotrexate-mtx-in-patients-with-rheumatoid-art/> Accessed January 2018
16. Emery P, Vencovský J, Sylwestrzak A et al (2016) THU0150 Long-Term Safety and Efficacy of SB4 (Etanercept Biosimilar) in Patients with Rheumatoid Arthritis: Comparison between Continuing SB4 and Switching from Etanercept Reference Product To SB4. Annals of the Rheumatic Diseases 75:236. <https://doi.org/10.1136/annrheumdis-2016-eular.3137>
17. Emery P, Vencovský J, Sylwestrzak A et al (2016) Additional Efficacy Results of SB4 (Etanercept Biosimilar) up to Week 100: Comparison Between Continuing SB4 and Switching from Reference Etanercept (Enbrel®) to SB4 [abstract]. Arthritis & Rheumatology 68(suppl 10). <http://acrabstracts.org/abstract/additional-efficacy-results-of-sb4-etanercept-biosimilar-up-to-week-100-comparison-between-continuing-sb4-and-switching-from-reference-etanercept-enbrel-to-sb4/> Accessed July 2017
18. Kivitz AJ, Papp K, Devani A et al (2016) Randomized, Double-Blind Study Comparing Chs-0214 with Etanercept (Enbrel) in Patients with Psoriasis and Psoriatic Arthritis [abstract]. Arthritis & Rheumatology 68(suppl 10). <http://acrabstracts.org/abstract/randomized-double-blind-study-comparing-chs-0214-with-etanercept-enbrel-in-patients-with-psoriasis-and-psoriatic-arthritis/> Accessed July 2017
19. Lee Y, Shin D, Kim Y et al (2015) SAT0176 A Phase I Pharmacokinetic Study Comparing SB4, An Etanercept Biosimilar, and Etanercept Reference Product (Enbrel®) in Healthy Male Subjects. Annals of the Rheumatic Diseases 74:718. <https://doi.org/10.1136/annrheumdis-2015-eular.2671>
20. Vencovský J, Sylwestrzak A, Leszczyński P et al (2015) FRI0128 A Phase III Randomised, Double-Blind Clinical Study Comparing SB4, An Etanercept Biosimilar, With Etanercept Reference Product (Enbrel®) in Patients with Moderate to Severe Rheumatoid Arthritis Despite Methotrexate Therapy (24-Week Results). Annals of the Rheumatic Diseases 74:467–468. <https://doi.org/10.1136/annrheumdis-2015-eular.1220>
21. Vencovsky J, Sylwestrzak A, Leszczyñski P et al (2015) A Phase III, Randomized, Double-Blind Clinical Study Comparing SB4, an Etanercept Biosimilar, with Etanercept Reference Product (Enbrel®) in Patients with Moderate to Severe Rheumatoid Arthritis Despite Methotrexate Therapy (52-week Results) [abstract]. Arthritis & Rheumatology 67(suppl 10). <http://acrabstracts.org/abstract/a-phase-iii-randomized-double-blind-clinical-study-comparing-sb4-an-etanercept-biosimilar-with-etanercept-reference-product-enbrel-in-patients-with-moderate-to-severe-rheumatoid/> Accessed July 2017
22. Vencovsky J, Emery P, Keystone E et al (2017) OP0204 Impact of anti-drug antibody and injection site reaction on efficacy: 24-week results from a phase iii study comparing SB4 (etanercept biosimilar) with reference etanercept in patients with rheumatoid arthritis. Annals of the Rheumatic Diseases 76:137. <https://doi.org/10.1136/annrheumdis-2017-eular.3043>
23. Von Richter O et al (2017) GP2015, a proposed etanercept biosimilar: Pharmacokinetic similarity to its reference product and comparison of its autoinjector device with prefilled syringes. British Journal of Clinical Pharmacology 83(4):732–741. <https://doi.org/10.1111/bcp.13170>.
24. Choe J, Smolen J, Keystone E et al (2016) THU0140 Efficacy and Safety Analysis by Overall Anti-Drug Antibody Results Up To Week 30 in Patients with Rheumatoid Arthritis Treated with Sb2 (An Infliximab Biosimilar) or Infliximab Reference Product in Phase III Study. Annals of the Rheumatic Diseases 75:232. <https://doi.org/10.1136/annrheumdis-2016-eular.3359>
25. Choe JY, Prodanovic N, Niebrzydowski J et al (2015) A Randomized, Double-Blind, Phase III Study Comparing SB2, an Infliximab Biosimilar, to the Infliximab Reference Product (Remicade®) in Patients with Moderate to Severe Rheumatoid Arthritis Despite Methotrexate Therapy: 54-Week Results [abstract]. Arthritis & Rheumatology 67(suppl 10). <http://acrabstracts.org/abstract/a-randomized-double-blind-phase-iii-study-comparing-sb2-an-infliximab-biosimilar-to-the-infliximab-reference-product-remicade-in-patients-with-moderate-to-severe-rheumatoid-arthritis-despi/> Accessed January 2018.
26. Choe J, Prodanovic N, Niebrzydowski J et al (2017) A randomised, double-blind, phase III study comparing SB2, an infliximab biosimilar, to the infliximab reference product Remicade in patients with moderate to severe rheumatoid arthritis despite methotrexate therapy. Annals of the Rheumatic Diseases 76:58–64. <https://doi.org/10.1136/annrheumdis-2015-207764>
27. Denisov L, Gordeev I, Mazurov V et al (2017) FRI0208 Comparison of efficacy, safety and pharmacokinetics of infliximab biosimilar (BCD-055) and innovator infliximab. Annals of the Rheumatic Diseases 76:560–561. <https://doi.org/10.1136/annrheumdis-2017-eular.4723>
28. Jorgensen K, Olsen I, Goll G et al (2017) Switching from originator infliximab to biosimilar CT-P13 compared with maintained treatment with originator infliximab (NOR-SWITCH): a 52-week, randomised, double-blind, non-inferiority trial. The Lancet 389:2304–2316. <http://dx.doi.org/10.1016/S0140-6736(17)30068-5>.
29. Global Alliance of Patient Access. White paper on NOR-SWITCH: What will Norway's infliximab switching study tell us about the safety of switching patients from one biologic medicine to a biosimilar? <http://gafpa.org/wp-content/uploads/GAfPA_Norswitch_Sept.2016.pdf> Accessed November 2017
30. Kaur P, Chow V, Zhang N et al (2016) Pharmacokinetic Similarity of ABP 710 Relative to Infliximab: Results from a Randomized, Single-Blind, Single-Dose, Parallel Group Study in Healthy Subjects [abstract]. Arthritis & Rheumatology 68(suppl 10). <http://acrabstracts.org/abstract/pharmacokinetic-similarity-of-abp-710-relative-to-infliximab-results-from-a-randomized-single-blind-single-dose-parallel-group-study-in-healthy-subjects/> Accessed July 2017
31. Kay J, Wyand M, Chandrashekara S et al (2014) BOW015, a biosimilar infliximab, in patients with active rheumatoid arthritis on stable methotrexate doses: 54-week results of a randomized, double-blind, active comparator study. Arthritis & Rheumatology 66. <http://acrabstracts.org/abstract/bow015-a-biosimilar-infliximab-in-patients-with-active-rheumatoid-arthritis-on-stable-methotrexate-doses-54-week-results-of-a-randomized-double-blind-active-comparator-study/> Accessed July 2017
32. Castañeda-Hernández G, Wyand M, Lassen C et al (2016) THU0133 Statistical Assessment across Multiple Early Time Points Validates Biosimilarity of BOW015 To Reference Infliximab in Patients with Active Rheumatoid. Arthritis on Stable Methotrexate Doses. Annals of the Rheumatic Diseases 75:228–9. <https://doi.org/10.1136/annrheumdis-2016-eular.2740>
33. Park SH, Kim YH, Lee JH et al (2015) Post-marketing study of biosimilar infliximab (CT-P13) to evaluate its safety and efficacy in Korea. Expert Reviews in Gastroenterology and Hepatology 9(Suppl 1):35–44. <https://doi.org/10.1586/17474124.2015.1091309>.
34. Park W, Hrycaj P, Jeka S et al (2013) A randomised, double-blind, multicentre, parallel-group, prospective study comparing the pharmacokinetics, safety, and efficacy of CT-P13 and innovator infliximab in patients with ankylosing spondylitis: the PLANETAS study. Annals of the Rheumatic Diseases 72:1605–1612. <https://doi.org/10.1136/annrheumdis-2012-203091>.
35. Park W, Yoo D, Miranda P et al (2017) Efficacy and safety of switching from reference infliximab to CT-P13 compared with maintenance of CT-P13 in ankylosing spondylitis: 102-week data from the PLANETAS extension study. Annals of the Rheumatic Diseases 76:346–54. <https://doi.org/10.1136/annrheumdis-2015-208783>.
36. Shin D, Kim Y, Kim Y et al (2015) SAT0144 A Phase I Pharmacokinetic Study Comparing SB2, An Infliximab Biosimilar, And Infliximab Reference Product (Remicade®) in Healthy Subjects. Annals of the Rheumatic Diseases 74:703. <https://doi.org/10.1136/annrheumdis-2015-eular.1265>.
37. Smolen JS, Choe JY, Prodanovic N et al (2016) Comparable Safety and Immunogenicity and Sustained Efficacy after Transition to SB2 (An Infliximab Biosimilar) Vs Ongoing Reference Infliximab (Remicade®) in Patients with Rheumatoid Arthritis: Results of Phase III Transition Study [abstract]. Arthritis & Rheumatology 68(suppl 10). <http://acrabstracts.org/abstract/comparable-safety-and-immunogenicity-and-sustained-efficacy-after-transition-to-sb2-an-infliximab-biosimilar-vs-ongoing-reference-infliximab-remicade-in-patients-with-rheumatoid-arthrit/> Accessed July 2017
38. Tanaka Y, Yamanaka H, Takeuchi T et al (2017) Safety and efficacy of CT-P13 in Japanese patients with rheumatoid arthritis in an extension phase or after switching from infliximab. Modern Rheumatology 27:237–245. <https://doi.org/10.1080/14397595.2016.1206244>
39. Udata C et al (2014) Immunogenicity Assessment of PF-06438179, a Potential Biosimilar to Infliximab, in Healthy Volunteers. Arthritis & Rheumatology 66. <http://acrabstracts.org/abstract/immunogenicity-assessment-of-pf-06438179-a-potential-biosimilar-to-infliximab-in-healthy-volunteers/> Accessed July 2017
40. Udata C, Yin D, Cai C et al (2015) SAT0142 Immunogenicity Assessment of PF-06438179, A Potential Biosimilar to Infliximab, In Healthy Volunteers. Annals of the Rheumatic Diseases 74:702. <https://doi.org/10.1136/annrheumdis-2015-eular.4209>
41. Yoo DH, Hrycaj P, Miranda P et al (2013) A randomised, double-blind, parallel-group study to demonstrate equivalence in efficacy and safety of CT-P13 compared with innovator infliximab when coadministered with methotrexate in patients with active rheumatoid arthritis: the PLANETRA study. Annals of the Rheumatic Diseases 72:1613–1620. <https://doi.org/10.1136/annrheumdis-2012-203090>
42. Yoo DH, Prodanovic N, Jaworski J et al (2017) Efficacy and safety of CT-P13 (biosimilar infliximab) in patients with rheumatoid arthritis: comparison between switching from reference infliximab to CT-P13 and continuing CT P13 in the PLANETRA extension study. Annals of the Rheumatic Diseases 76:355–363. <https://doi.org/10.1136/annrheumdis-2015-208786>.
43. Yoo DH et al (2014) Abstract number 1509. A Randomized, Double-Blind, Three-Arm, Parallel Group, Single-Dose Study to Compare the Pharmacokinetics, Safety, and Tolerability of Three Formulations of Infliximab (CT-P13, EU-sourced Infliximab and US-sourced Infliximab) in Healthy Volunteers. Arthritis & Rheumatology 66. <http://acrabstracts.org/abstract/a-randomized-double-blind-three-arm-parallel-group-single-dose-study-to-compare-the-pharmacokinetics-safety-and-tolerability-of-three-formulations-of-infliximab-ct-p13-eu-sourced-infliximab-an/> Accessed July 2017.
44. Becker JP et al (2014) Abstract number 1502. A Phase I Trial Comparing PF-05280586 (A Potential Biosimilar) and Rituximab in Subjects with Active Rheumatoid Arthritis. Arthritis & Rheumatology 66. <http://acrabstracts.org/abstract/a-phase-i-trial-comparing-pf-05280586-a-potential-biosimilar-and-rituximab-in-subjects-with-active-rheumatoid-arthritis/> Accessed July 2017
45. Eremeeva A, Chernyaeva E, Ivanov R et al (2016) FRI0224 Comparison of Efficacy and Safety of Rituximab Biosimilar, BCD-020, and Innovator Rituximab in Patients with Active Rheumatoid Arthritis Refractory To TNFA Inhibitors. Annals of the Rheumatic Diseases 75:513–514. <https://doi.org/10.1136/annrheumdis-2016-eular.1715>.
46. Nasonov E, Mazurov V, Plaksina T et al (2016) Interchangeability of Innovator Rituximab and Its Biosimilar: Results from International Controlled Comparative 1-Year Study in Patients with Active Rheumatoid Arthritis [abstract]. Arthritis & Rheumatology 68(suppl 10). <http://acrabstracts.org/abstract/interchangeability-of-innovator-rituximab-and-its-biosimilar-results-from-international-controlled-comparative-1-year-study-in-patients-with-active-rheumatoid-arthritis/> Accessed July 2017
47. Smolen J, Scheinberg M, Tony H et al (2016) FRI0222 Pharmacokinetics, Pharmacodynamics, Safety and Efficacy of Proposed Rituximab Biosimilar (GP2013) vs. EU-Approved Rituximab (RTX) in Patients with Rheumatoid Arthritis: Results from A Randomized Controlled Trial (GP13-201) over 52 Weeks. Annals of the Rheumatic Diseases 75:512–513. <https://doi.org/10.1136/annrheumdis-2016-eular.1578>
48. Suh CH, Berrocal Kasay A, Chalouhi El-Khouri E et al (2016) Pharmacokinetics and Safety of Three Formulations of Rituximab (CT-P10, US-sourced Innovator Rituximab and EU-sourced Innovator Rituximab) in Patients with Rheumatoid Arthritis: Results from Phase 3 Randomized Controlled Trial over 24 Weeks [abstract]. Arthritis & Rheumatology 68(suppl 10). <http://acrabstracts.org/abstract/pharmacokinetics-and-safety-of-three-formulations-of-rituximab-ct-p10-us-sourced-innovator-rituximab-and-eu-sourced-innovator-rituximab-in-patients-with-rheumatoid-arthritis-results-from-phase-3-r/> Accessed July 2017
49. Suh C, Khouri ECE, Miranda P et al (2017) SAT0146 Randomised double-blind study shows comparable long-term efficacy and safety between rituximab biosimilar CT-P10 and innovator rituximab in patients with rheumatoid arthritis: 48-week results. Annals of the Rheumatic Diseases 76:824–825. <https://doi.org/10.1136/annrheumdis-2017-eular.6553>
50. Yin D, Becker J, Melia L et al (2014) FRI0309 A Phase I Pharmacokinetics TRIAL Comparing PF-05280586 (A Potential Biosimilar) and Rituximab in Subjects with Active Rheumatoid Arthritis with Active Disease in TNF Failures (Reflections B328-01). Annals of the Rheumatic Diseases 73:497. <https://doi.org/10.1136/annrheumdis-2014-eular.5444>.
51. Yoo DH, Park W, Suh CH et al (2015) Abstract number 1675. Efficacy and Safety of Switched CT-P10 from Innovator Rituximab Compared to Those of Maintained CT-P10 in Patients with Rheumatoid Arthritis up to 56 Weeks [abstract]. Arthritis & Rheumatology. 67 (suppl 10). <http://acrabstracts.org/abstract/efficacy-and-safety-of-switched-ct-p10-from-innovator-rituximab-compared-to-those-of-maintained-ct-p10-in-patients-with-rheumatoid-arthritis-up-to-56-weeks/> Accessed July 2017
52. Yoo DH, Park W, Suh CH et al (2015) Abstract number 2058. Efficacy and Safety of Rituximab Biosimilar Candidate (CT-P10) and Innovator Rituximab in Patients with Rheumatoid Arthritis: Results from Phase I Randomized Controlled Trial over 72 Weeks [abstract]. Arthritis & Rheumatology 67(suppl 10). <http://acrabstracts.org/abstract/efficacy-and-safety-of-rituximab-biosimilar-candidate-ct-p10-and-innovator-rituximab-in-patients-with-rheumatoid-arthritis-results-from-phase-i-randomized-controlled-trial-over-72-weeks/> Accessed July 2017
53. Yoo DH, Bozic Majstorovic L et al Abstract number 1635. Efficacy and Safety of CT-P10, Rituximab Biosimilar Candidate, and Innovator Rituximab in Patients with Rheumatoid Arthritis: Results from Phase 3 Randomized Controlled Trial over 24 Weeks [abstract]. Arthritis & Rheumatology 2016;68 (suppl 10). <http://acrabstracts.org/abstract/efficacy-and-safety-of-ct-p10-rituximab-biosimilar-candidate-and-innovator-rituximab-in-patients-with-rheumatoid-arthritis-results-from-phase-3-randomized-controlled-trial-over-24-weeks/> Accessed July 2017
54. Agarwal M, Shivpuri A, Mittal S et al (2016) Abstract number 390. Use, Safety and Efficacy of Zrc 3197, a Biosimilar Candidate for Reference Adalimumab (Humira) from a Tertiary Pediatric Rheumatology Centre in India [abstract]. Arthritis & Rheumatology 68(suppl 10). <http://acrabstracts.org/abstract/use-safety-and-efficacy-of-zrc-3197-a-biosimilar-candidate-for-reference-adalimumab-humira-from-a-tertiary-pediatric-rheumatology-centre-in-india/> Accessed July 2017
55. Bandyopadhyay S, Ray A, Sarkar R et al (2017) AB0705 Continued effectiveness of a biosimilar adalimumab after stoppage of initial treatment in patients with ankylosing spondylitis. Annals of the Rheumatic Diseases 76:1300. <https://doi.org/10.1136/annrheumdis-2017-eular.3825>.
56. Kaushik V, Apparao S (2017) FRI0209 Real-life safety profile of biosimilar adalimumab in patients with inflammatory arthritic conditions. Annals of the Rheumatic Diseases 76:561. <https://doi.org/10.1136/annrheumdis-2017-eular.2357>.
57. Emery P, Vencovsky J, Sylwestrzak A et al (2017) A phase III randomised, double-blind, parallel-group study comparing SB4 with etanercept reference product in patients with active rheumatoid arthritis despite methotrexate therapy. Annals of the Rheumatic Diseases76(1):51–57. <https://doi.org/10.1136/annrheumdis-2015-207588>.
58. Holroyd C, Wallis D, Bennett S et al (2017) AB0377 Switching from bio-original etanercept to biosimilar etanercept SB4: patient acceptability and outcomes in the real world. Annals of the Rheumatic Diseases 76:1180. <https://doi.org/10.1136/annrheumdis-2017-eular.3672>.
59. Shivpuri A, Mittal S, Agarwal M et al (2016) A Single Centre Experience from India on the Safety and Efficacy of Cipla Etanercept and Intas Etanercept and Its Comparison with Reference Etanercept(Enbrel) in Children with JIA [abstract]. Arthritis & Rheumatology 68(suppl 10). <http://acrabstracts.org/abstract/a-single-centre-experience-from-india-on-the-safety-and-efficacy-of-cipla-etanercept-and-intas-etanercept-and-its-comparison-with-reference-etanerceptenbrel-in-children-with-jia/> Accessed July 2017
60. Abdalla A, Byrne NE, Conway R et al (2016) THU0120. Long term safety and efficacy of biosimilar infliximab among patients with inflammatory arthritis switched from reference product. Annals of the Rheumatic Diseases 75(suppl2):223. <https://doi.org/10.1136/annrheumdis-2016-eular.2924>
61. Abdalla A et al (2017) Long-term safety and efficacy of biosimilar infliximab among patients with inflammatory arthritis switched from reference product. Rheumatology: Research and Reviews 9:29–35. <https://doi.org/10.2147/oarrr.s124975>
62. Agrawal I, Roy A, Kiran R et al (2017) AB0690 Clinical experience with infliximab biosimilar (BOW015) in ankylosing spondylitis- efficacy and safety analysis from an Indian perspective. Annals of the Rheumatic Diseases 76:1295. <https://doi.org/10.1136/annrheumdis-2017-eular.5865>
63. Akrout W, Bosycot A, Levet-Labry R et al (2017) AB0708 Transition from ongoing infliximab reference product to its biosimilar: can we talk about a failure? Annals of the Rheumatic Diseases 2017;76:1301–1302. <https://doi.org/10.1136/annrheumdis-2017-eular.4823>.
64. Avouac J, Molto A, Abitbol V et al (2017) SAT0163 Systematic switch from innovator infliximab to biosimilar infliximab in inflammatory rheumatic diseases in daily clinical practice: the experience of Cochin Hospital, Paris, France. Annals of the Rheumatic Diseases 76:831–832. <https://doi.org/10.1136/annrheumdis-2017-eular.3791>
65. Batticciotto A, Parisi S, Gobbi FL et al (2016) SAT0381 Safety and efficacy of switching from innovator to biosimilar infliximab in patients affected by spondyloarthritis. A 6-month observational study. Annals of the Rheumatic Diseases 75:806. <https://doi.org/10.1136/annrheumdis-2016-eular.5115>.
66. Codreanu C, Sirova K, Jarosova K et al (2016) Abstract number 621. Effectiveness and Safety of CT- P13 (Biosimilar Reference Infliximab) in a Real-Life Setting in 151 Patients with Rheumatoid Arthritis and Ankylosing Spondylitis: A Mid-Term Interim Analysis [abstract]. Arthritis & Rheumatology 68(suppl 10). <http://acrabstracts.org/abstract/effectiveness-and-safety-of-ct-p13-biosimilar-reference-infliximab-in-a-real-life-setting-in-151-patients-with-rheumatoid-arthritis-and-ankylosing-spondylitis-a-mid-term-interim-analysis/> Accessed July 2017
67. Glintborg B, Juul Sørensen I, Jensen DV et al (2016) Abstract number 951. A Nationwide Non-Medical Switch from Originator to Biosimilar Infliximab in Patients with Inflammatory Arthritis. Eleven Months’ Clinical Outcomes from the Danbio Registry [abstract]. Arthritis & Rheumatology 68(suppl 10). <http://acrabstracts.org/abstract/a-nationwide-non-medical-switch-from-originator-to-biosimilar-infliximab-in-patients-with-inflammatory-arthritis-eleven-months-clinical-outcomes-from-the-danbio-registry/> Accessed July 2017
68. Glintborg B, Kringelbach TM, Høgdall E et al (2016) Abstract number 1997. Non-Medical Switch from Originator to Biosimilar Infliximab in Patients with Inflammatory Arthritis – Impact on s-Infliximab and Antidrug-Antibodies. Results from the Danish Rheumatologic Biobank and the Danbio Registry [abstract]. Arthritis & Rheumatology 68(suppl 10). <http://acrabstracts.org/abstract/non-medical-switch-from-originator-to-biosimilar-infliximab-in-patients-with-inflammatory-arthritis-impact-on-s-infliximab-and-antidrug-antibodies-results-from-the-danish-rheumatologic-biobank-and/> Accessed July 2017
69. Glintborg B, Juul Sørensen I, Vendelbo Jensen D et al (2016) OP0225 Three Months' Clinical Outcomes from A Nationwide Non-Medical Switch from Originator To Biosimilar Infliximab in Patients with Inflammatory Arthritis. Results from The Danbio Registry. Annals of the Rheumatic Diseases 75:142. <https://doi.org/10.1136/annrheumdis-2016-eular.1785>.
70. Joseph J, Solonos M, Michaelides M et al (2017) AB1093 Biosimilar infliximab in rheumatology practice — the Cyprus experience*.* Annals of the Rheumatic Diseases 76:1437. <https://doi.org/10.1136/annrheumdis-2017-eular.4200>
71. Presberg Y, Foltz V, L'Amour C et al (2017) THU0646 Interchangeability from infliximab originator to infliximab biosimilar: efficacy and safety in a prospective observational study on 89 patients. Annals of the Rheumatic Diseases 76:450. <https://doi.org/10.1136/annrheumdis-2017-eular.6586>
72. Rubio E, Ruiz A, Lόpez J et al (2016) AB0310 Prospective Study of 78 Patients Treated with Infliximab Biosimilar Remsima®. Annals of the Rheumatic Diseases 75:1006. <https://doi.org/10.1136/annrheumdis-2016-eular.5688>
73. Sokka T, Kautiainen H (2015) SAT0174 Clinical Experience with Infliximab Biosimilar – Switch from Remicade. Annals of the Rheumatic Diseases 74:717. <https://doi.org/10.1136/annrheumdis-2015-eular.2151>
74. Sung YK, Cho SK, Won S et al (2015) Abstract number 1221. Characteristics and Outcomes of RA Patients Who Start Biosimilar Infliximab in South Korea [abstract]. Arthritis & Rheumatology 67(suppl 10). <http://acrabstracts.org/abstract/characteristics-and-outcomes-of-ra-patients-who-start-biosimilar-infliximab-in-south-korea/> Accessed July 2017
75. Tweehuysen L, van den Bemt BJF, van Ingen IL et al (2016) Abstract number 627. Clinical and Immunogenicity Outcomes after Switching Treatment from Innovator Infliximab to Biosimilar Infliximab in Rheumatic Diseases in Daily Clinical Practice [abstract]. Arthritis & Rheumatology 68(suppl 10). <http://acrabstracts.org/abstract/clinical-and-immunogenicity-outcomes-after-switching-treatment-from-innovator-infliximab-to-biosimilar-infliximab-in-rheumatic-diseases-in-daily-clinical-practice/> Accessed July 2017
76. Yazici Y, Xie L, Ogbomo A et al (2016) A descriptive analysis of real-world treatment patterns in a Turkish rheumatology population that continued innovator infliximab (Remicade) therapy or switched to biosimilar infliximab. Arthritis & Rheumatology 68(suppl10). <http://acrabstracts.org/abstract/a-descriptive-analysis-of-real-world-treatment-patterns-in-a-turkish-rheumatology-population-that-continued-innovator-infliximab-remicade-therapy-or-switched-to-biosimilar-infliximab/> Accessed July 2017

## Table 4: Key messages from rheumatology society position papers on biosimilars

| **Subject** | **Position statements** | **Organizations that support the position** |
| --- | --- | --- |
| **Drug selection** | The less expensive drug is a reasonable first therapeutic choice in naïve patients. | Agenzia Italiana del Farmaco [1] |
|  | Drug choice should be based on individual risk benefit profile, not just cost, and is based on the decision of the prescribing physician. | Portuguese Society of Rheumatology [2]  German Rheumatism League [3]  Spanish Society of Rheumatology [4]  American College of Rheumatology [5]  British Society for Rheumatology [6] |
| **Substitution and interchangeability** | Automatic substitution is unacceptable. Substitution can only be carried out after consent of the prescribing physician. | Colegio Mexicano de Reumatología [7]  Portuguese Society of Rheumatology [2]  German Rheumatism League [3]  German Society of Rheumatology [8]  National Rheumatoid Arthritis Society [9]  Spanish Society of Rheumatology [4]  American College of Rheumatology [5]  British Society for Rheumatology [6] |
|  | When biosimilars have the same INN, medicines must be prescribed by brand name. | Portuguese Society of Rheumatology [2]  Spanish Society of Rheumatology [4]  American College of Rheumatology [5] |
|  | All biologics and biosimilars must be prescribed by Brand name and not INN. | National Rheumatoid Arthritis Society [9]  American College of Rheumatology [5]  British Society for Rheumatology [6] |
| **Switching** | Switching between biosimilars and the original product must be performed only upon consent of the attending physician. | American College of Rheumatology [5]  Colegio Mexicano de Reumatología [7]  Portuguese Society of Rheumatology [2]  European Crohn’s and Colitis Organization [10]  German Society of Rheumatology [8]  Italian Society of Rheumatology [1]  National Rheumatoid Arthritis Society [9]  Spanish Society of Rheumatology [4]  British Society for Rheumatology [6] |
|  | Switching requires informed consent  of the patient. | Portuguese Society of Rheumatology [2]  National Rheumatoid Arthritis Society [9]  British Society for Rheumatology [6]  European Crohn’s and Colitis Organization [10] |
|  | Allow six months on biologic before switching. | Portuguese Society of Rheumatology [2] |
|  | Label should clearly indicate whether a biologic is interchangeable with the reference product. | American College of Rheumatology [5] |
| **Extrapolation of indications** | Extrapolation of indications approved for the originator drug to completely different diseases and age groups that are not based on adequate pre-clinical,  safety and efficacy data (ideally phase I and phase III trials) should not be performed. | Portuguese Society of Rheumatology [2] |
|  | Extrapolation must be decided on a  case-by-case basis or requires additional clinical data. | Spanish Society of Rheumatology [4]  American College of Rheumatology [5] |
|  | Clinical studies of equivalence in the most sensitive indication can provide the basis for extrapolation. | European Crohn’s and Colitis Organization [10]  Egypt Drug Authority [12] |
|  | Data demonstrating safety parameters for the intended indication are the same as reference product. | German Rheumatism League [8] |
| **Immunogenicity** | Immunogenicity must be adequately  assessed. | Portuguese Society of Rheumatology [2]  Egypt Drug Authority [11]  European Crohn’s and Colitis Organization [10]  American College of Rheumatology [5] |
| **Pharmacovigilance** | Robust pharmacovigilance strategies must be used. | American College of Rheumatology [5]  Colegio Mexicano de Reumatología [7]  Portuguese Society of Rheumatology [2]  Spanish Society of Rheumatology [4]  British Society for Rheumatology [6]  European Crohn’s and Colitis Organization [10]  Egypt Drug Authority [11]  , |
|  | Patient registries and clinical databases are important sources of data  concerning rare adverse events. | American College of Rheumatology [5]  Spanish Society of Rheumatology [4]  British Society for Rheumatology [6]  European Crohn’s and Colitis Organization [10] |
|  | Brand name, batch number & date of administration must be added to national registry. | Portuguese Society of Rheumatology [2]  British Society for Rheumatology [6] |

1. Agenzia Italiana del Farmaco (2013) AIFA Position Paper. <http://www.agenziafarmaco.gov.it/sites/default/files/AIFA_POSITION_PAPER_FARMACI_BIOSIMILARI.pdf> Accessed November 2017
2. Fonseca JE, Gonçalves J, Araújo F et al (2014) The Portuguese Society of Rheumatology position paper on the use of biosimilars. Acta Reumatológica Portuguesa. 39(1):60–71
3. German Rheumatism League (2014) Positionierung der Deutschen Rheuma-Liga Bundesverband e.V.zur Einfuhrung von Biosimilars in Deutschland. Rheuma-liga. <https://www.rheuma-liga.de/biosimilars/> Accessed November 2017
4. Abad Hernández MÁ, Andreu JL, Caracuel Ruiz MÁ et al (2015) Position paper from the Spanish Society of Rheumatology on biosimilar drugs. Reumatología Clínica. 11(5):269–278. <https://doi.org/10.1016/j.reumae.2015.03.012>.
5. American College of Rheumatology (2016) Position statement on biosimilars. Rheumatology. <https://www.rheumatology.org/Portals/0/Files/Biosimilars-Position-Statement.pdf>. Accessed November 2017
6. British Society for Rheumatology (2017) Position statement on biosimilar medicines <https://nass.co.uk/download/589865499d2e4> Accessed November 2017
7. Xibile D, Carillo S, Huerta-Sil G et al (2017) Current state of biosimilars in Mexico: The position of the Mexican College of Rheumatology. *Reumatologica Clinica* 2013;9:113–116. <https://doi.org/10.1016/j.reumae.2012.11.003>
8. Lorenz HM et al (2014) Einführung und Gebrauch von „biosimilars“ in der Therapie entzündlich-rheumatischer Erkrankungen. Zeitschrift für Rheumatologie 73(9):784–786. <https://doi.org/10.1007/s00393-014-1505-x>.
9. National Rheumatoid Arthritis Society (2017) NRAS position paper on biosimilar medicines. <https://www.nras.org.uk/data/files/Publications/NRAS%20Revised%20position%20paper%20Final%206.6.16.pdf> Accessed November 2017
10. Danese S et al (2016) ECCO Position Statement on the Use of Biosimilars for Inflammatory Bowel Disease—An Update*.* Journal of Crohn's and Colitis 11(1):26–34. <https://doi.org/10.1093/ecco-jcc/jjw198>.
11. Egypt Drug Authority (2015) Guidelines for Registration of Biosimilar products in Egypt. <http://www.eda.mohp.gov.eg/Files/774_Guidelines%20For%20Registration%20of%20Biosimilar%20Products%20in%20Egypt.pdf>. Accessed January 2018.
